# Training the trainers: improving the quality of education delivered to paramedics through a simulation-debrief model

**DOI:** 10.29045/14784726.2023.3.7.4.51

**Published:** 2023-03-01

**Authors:** Pascale Avery, Charlotte Thompson, Philip Cowburn

**Affiliations:** South Western Ambulance Service NHS Foundation Trust; South Western Ambulance Service NHS Foundation Trust; South Western Ambulance Service NHS Foundation Trust

**Keywords:** paramedic education, sim-debrief, simulation, simulation-debrief, training

## Abstract

**Introduction:**

Education and training delivered within ambulance services is vital to clinicians maintaining competence, confidence and currency. Simulation and debrief in medical education aims to imitate clinical experience and provide real-time feedback. The South Western Ambulance Service NHS Foundation Trust employs senior doctors in their learning and development (L&D) team to support the development of ‘train the trainer’ courses for L&D officers (LDOs). This short report of a quality improvement initiative describes the implementation and evaluation of a simulation-debrief model of paramedic education.

**Methods:**

A quality improvement design was adopted. The train the trainer scenarios for simulation-debrief were designed and written following the trust’s training needs analysis by the L&D team. The course ran for two days, and each scenario was facilitated by faculty experienced in simulation (both doctors and paramedics). Low-fidelity mannequins and standard ambulance training kit was used (including response bags, training monitor and defibrillator). Participants’ pre- and post-scenario self-reported confidence scores were recorded, and qualitative feedback requested. Numerical data were analysed, and collated into graphs using Excel. Thematic analysis of comments was used to present qualitative themes. The SQUIRE 2.0 checklist for reporting quality improvement initiatives was used to frame this short report.

**Results:**

Forty-eight LDOs attended across three courses. All participants reported improved confidence scores in the clinical topic covered after each simulation-debrief scenario, with a minority reporting equivocal scores. Formal qualitative feedback from participants indicated an overwhelmingly positive response to the introduction of simulation-debrief as an education method, and a move away from summative, assessment-based training. The positive value of a multidisciplinary faculty was also reported.

**Conclusion:**

The simulation-debrief model of paramedic education represents a move away from the use of didactic teaching and ‘tick box’-style assessments in previous train the trainer courses. The introduction of simulation-debrief teaching methodology has had a positive impact on paramedics’ confidence in the selected clinical topics, and is seen by LDOs as an effective and valuable education method.

## Introduction

Education and training delivered within ambulance services to front-line clinical staff is of paramount importance. It is vital that clinicians’ skills and knowledge are up to date so that they are able to practise person-centred, safe and effective care. This requires operational staff to engage in regular education and professional development to match the changing demands and complexity of pre-hospital emergency care.

The South Western Ambulance Service NHS Foundation Trust (SWASFT) employs 58 senior paramedics as trainers, called learning and development officers (LDOs), who play a crucial role in the delivery of training and education of all front-line clinical staff. One of their responsibilities is to deliver the annual development days to all clinical staff, with a focus on key clinical skills such as airway management and electrocardiogram interpretation.

In 2020, SWASFT employed two specialist doctors in emergency medicine with a background in medical education as part of the learning and development (L&D) team. The role included a redesign of the annual ‘train the trainer’ event for LDOs. The purpose of the ‘train the trainer’ events was to build confidence, train where needed and quality assure the LDOs in the material that they are expected to deliver at development day – as well as to introduce simulation-debrief as a new method of delivery. This report will describe the process of implementing the simulation-debrief-based training course for senior paramedic trainers. The aim of this study was to improve the quality of education delivered to LDOs, and subsequently the clinicians they train, through the ‘simulation-debrief’ model.

## Methods

### Study design and setting

SWASFT employs over 4000 operational staff serving an area of 10,000 square miles, with a population of over 5.5 million people and in receipt of ~23 million visitors each year. Education delivery in SWASFT historically consisted of ‘tick box’-style assessments. We sought to improve senior clinician confidence in educational material delivered trust-wide, using a simulation-debrief model. A quality improvement design was adopted (Smith et al., 2020).

### Participants

The participants in this quality improvement initiative were LDOs undertaking a bespoke train the trainer course designed to introduce simulation-debrief as a method of delivering education. LDOs will use simulation-debrief to deliver education to clinicians across SWASFT on their annual development days.

### Improvement initiative

#### Scenario development

Scenario topics were chosen based on the trust’s latest training needs analysis with risk assessment matrix scoring and learning identified from incident reviews. Specific learning outcomes were identified for each scenario. The scenarios were written collaboratively by learning and development managers (LDMs) and L&D doctors, then quality assured by independent specialist clinicians external to SWASFT (see example in Supplementary 1). The second day focused on paediatric scenarios (Table 1). A video introduction was produced for each scenario, to set the scene. Stations involved 20 minutes of simulation, and 40 minutes of structured debrief led by a minimum of two facilitators. Core learning objectives were built into the debrief.

**Table 1.** Scenarios covered over the two-day course.

Day 1 scenarios: Adult emergencies	Day 2 scenarios: Maternal and paediatric emergencies
Anaphylaxis	Shoulder dystocia
Asthma	Paediatric traumatic cardiac arrest
Adrenal crisis	Severe bronchiolitis
Complete heart block	Neonatal life support
Airway management	
Status epilepticus	

#### Faculty

A multidisciplinary faculty of scenario facilitators was chosen. All had experience of simulation-debrief medical education, and either pre-hospital experience or relevant specialist practice. Facilitators included senior doctors in emergency medicine, anaesthesia and critical care, and specialist paramedics in critical care.

#### Scenario format and equipment

Facilitators encouraged participants to immerse themselves fully in each scenario by emphasising the safe learning environment. Simple, low-fidelity, age-appropriate mannikins were used, and the standard SWASFT ambulance training kit was used (including response bags, training monitor and defibrillator). LDOs were divided into groups of five to six, with one pair taking the role of ‘first crew on scene’. Medical student volunteers were used to role play the part of the conscious patient. Once the ‘patient’ collapsed, the mannikin was used. Training defibrillators controlled by the facilitator were used, providing real-time feedback and avoiding a reliance on asking the facilitator for clinical information. Two members of each group were allocated to actively observe both clinical and non-clinical aspects. Remaining group members could be called on as back-up crews if required.

#### Debrief

Twice the amount of time was allocated for the group debrief as for the simulation, which took place immediately afterwards. Facilitator-guided debrief followed a formal structure, including a description of the scenario narrative, rationale behind key decisions and identification of areas that could be done differently (Sawyer et al., 2016). Those observing were encouraged to give constructive feedback, and questions generated from the scenario were discussed to increase depth of understanding.

#### Course implementation

The two-day course ran three times in early 2021. COVID-19 restrictions meant there were extra considerations in the planning and implementation. Masks were mandatory everywhere, personal protective equipment was worn in clinical scenarios and participants were placed in isolated ‘bubbles’ for the duration of the course.

### Quality measurement

With our objective of removing ‘tick box’-style assessments, there were no pre-determined assessment standards the LDOs had to meet throughout the course. A course evaluation was developed by LDMs and L&D doctors with clearly defined scoring criteria in line with recommendations (Santomauro et al., 2020). LDOs were asked to rate their confidence in each clinical topic on a scale of 1 to 10 pre and post each scenario, as well as to give qualitative feedback on the course. This anonymous evaluation was requested at each train the trainer course.

### Analysis

Numerical data were analysed, and collated into graphs using Excel, by CT. Qualitative thematic analysis of comments was used as a flexible approach to identifying, systematising and describing paramedic comments (Kiger & Varpio, 2020).

The SQUIRE 2.0 checklist for reporting quality improvement initiatives was used (Ogrinc et al., 2016).

## Results

**Figure 1:**
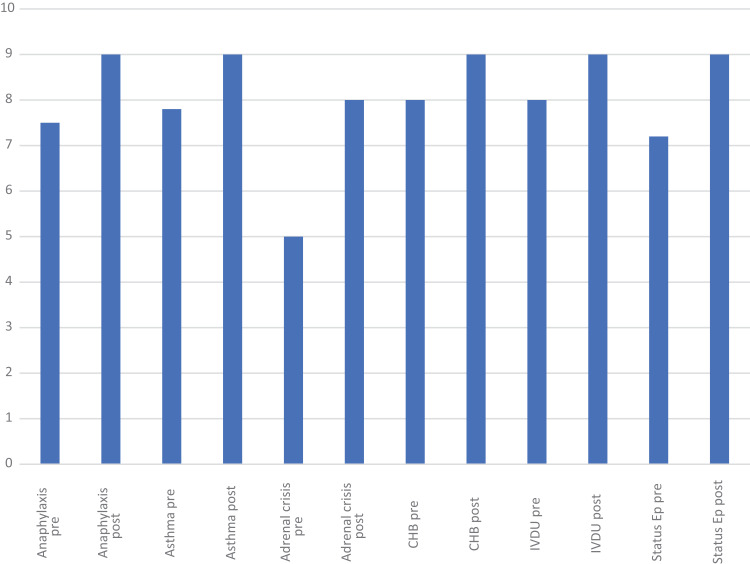
Figure 1. Average pre- and post-scenario self-reported confidence scores for each scenario from day one.

CHB: complete heart block; IVDU: intravenous drug use.

Forty-eight LDOs attended the train the trainer event across the three courses. Figure 1 shows the average (median) pre- and post-confidence scores for each scenario from day one of each course. Pre-scenario scores ranged from 5/10 to 8/10, and post-scenario scores from 8/10 to 9/10.

**Figure 2:**
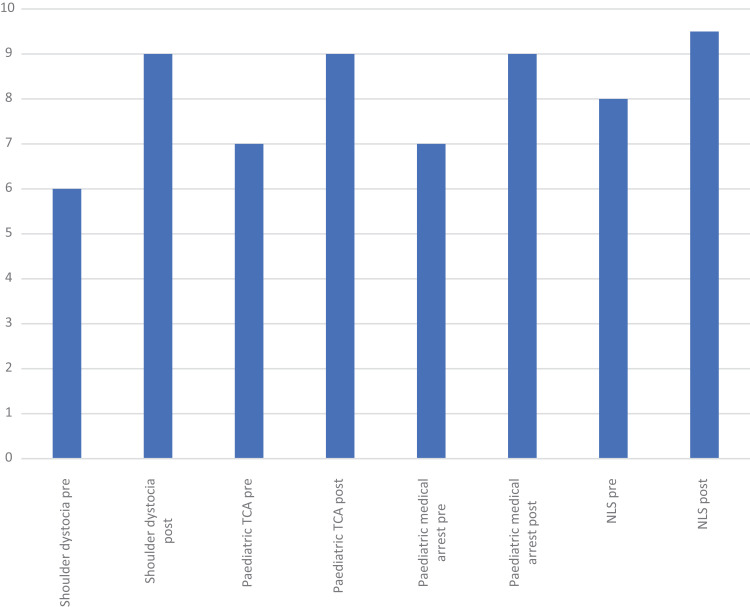
Figure 2. Average pre- and post-scenario self-reported confidence scores for each scenario from day two.

NLS: newborn life support; TCA: traumatic cardiac arrest.

Figure 2 shows the average (median) pre- and post-confidence scores for each scenario from day two, which were collected on the third run of the course in March.

### Scenario-based simulation

The most commonly occurring qualitative theme was a positive reaction to the introduction of simulation as a new way of training. Comment examples include:

*The sim and debrief model is a really effective teaching and learning model to employ.*

*Scenario-based learning cannot be underestimated, an excellent tool that I wish was consistently accessible for all staff.*

Another theme involved the move away from other education methods. Comment examples include:

*The fact it wasn’t an assessment made me happy to make mistakes and focus on areas I am less confident with. I learnt more on this TTT than any I have attended before.*

*Building learning outcomes into sim rather than PowerPoints.*

### Multidisciplinary faculty

The presence of a multidisciplinary faculty was a positive theme. Comment examples include:

*It was so helpful having feedback from the doctors and their various specialties. I felt it really helped boost knowledge and confidence.*

*Having doctors and specialist paramedics on each sim, and being able to discuss each element in detail.*

### Debrief

The use of structured debrief was a positive theme throughout the feedback, particularly the opportunity it afforded for group discussions. Comment examples include:

*Time to debrief was appreciated. Chance to ask those clinical questions.*

*The post-scenario discussions were great in drawing out the learning points.*

### Scope for improvement

Themes for improvement included equipment and uniform. Comment examples include:

*Better simulation kit to include what crews would have, e.g. MobiMed, etc.*

*Everyone wearing uniform rather than own clothes.*

## Discussion

### Simulation-debrief model

This is the first time that simulation-debrief has been used as a training method in SWASFT to facilitate train the trainer for the LDOs, to support and develop their own practice. We have demonstrated a clear improvement in confidence after each simulation across all scenarios on each running of the course (Figures 1 and 2). Thematic analysis of the qualitative feedback revealed an overwhelmingly positive response to simulation-debrief as an education method. Simulation-debrief has become a key part of education across the healthcare professions (McKelvin & McKelvin, 2020). Its use in paramedic undergraduate education is well described (McKenna et al., 2015); however, there is little high-quality evidence evaluating its use in ongoing paramedic training and education (McCallum et al., 2015; Sanfilippo et al., 2016). The main benefit of simulation is that it allows standardisation and consistent replication of patient conditions (McKenna et al., 2015).

### Debrief

An essential component of simulation is a structured debrief following the scenario. Debriefing maximises learning outcomes, helps identify erroneous decision making, and enables the scenario facilitator to reinforce key learning points, both clinical and non-technical (Bredmose et al., 2010). Literature supports debrief at least the length of simulation (Schertzer & Patti, 2022), with some articles suggesting two to three times the length of the scenario (Arafeh et al., 2010; Bae et al., 2019). A recent service evaluation of a UK ambulance trust reported improvements in identifying good practice and learning opportunities after using a validated structured debrief tool (Tierney, 2018).

### Assessment

One of the key differences of this train the trainer course was a desire on the part of senior SWASFT L&D staff to move away from a summative, assessment-based method of ongoing education for clinicians of all levels across the trust. Summative assessments as a benchmark of competence were generally feared by clinicians, and conferred negative feeling towards development days. Formative assessment has been shown to facilitate and enhance learning (Schuwirth & Van der Vleuten, 2011) and can be used to ensure acceptable levels of competence among medical trainees. Simulation-based assessment scores have been shown to correlate reliably with real clinical workplace-based assessment scores in Australian paramedic trainees (Tavares et al., 2014).

### Doctors supporting paramedic education

This is the first time to our knowledge that doctors have become embedded in the L&D team (or equivalent) within a UK ambulance trust, with the explicit aim of directly supporting senior paramedics responsible for education and appraisal of all trust clinical staff. Following an extensive search of the literature, no studies on the role of the doctor in paramedic post-qualification training were found, although there is emerging description of paramedics having a role in teaching elements of undergraduate medical curricula (Cassidy et al., 2013). Both L&D doctors are senior doctors in emergency medicine and as such are in a unique position to provide peer-to-peer learning for LDOs. Hierarchy is a potential barrier to interprofessional learning between paramedics and doctors (Mulholland et al., 2020), so this level of seniority is likely to be enough to provide meaningful insight into emergency care, but not so high as to be a barrier. The vast majority of senior doctors in emergency medicine are not pre-hospital experts; however, they can help provide a depth of understanding useful to senior paramedics. Senior doctors may be a useful asset to paramedic education by inspiring confidence in educators and quality assuring trust-wide teaching material. The train the trainer course delivered by doctors in the way we describe provides standardisation and consistency of approach in learning and development across a large ambulance service trust.

### Limitations

There were limitations in the delivery of this training course. The fidelity of monitoring equipment was low, as it was standard clinical kit rather than interactive monitoring designed for simulation. A number of ‘i-simulate’ mannikins have been purchased by the trust since train the trainer 2021 and will be used for next year’s course configured to match trust standard monitors to improve the fidelity of the simulation. Confidence levels pre and post scenario are easy data to gather practically, and they give some insight into the impact of the teaching method on the participants. However, an evaluation on the impact this has on LDOs’ ability to teach this material to clinical staff would be more meaningful.

## Conclusion

This is the first time that simulation-debrief has been used as a training method in SWASFT to facilitate train the trainer courses. Our quality improvement initiative has demonstrated a clear improvement in confidence after each simulation across all scenarios. Integrating doctors into paramedic education provides peer-to-peer support in education for senior paramedics responsible for the education and appraisal of a whole clinical pre-hospital workforce. The vast majority of doctors are not pre-hospital experts; however, they may help provide a depth of understanding useful to senior paramedics. By instilling confidence and quality assuring the material disseminated by paramedic educators across the trust, it provides standardisation and consistency of approach to the wider clinical workforce. Future evaluation should include the effectiveness of the sim-debrief model delivered trust-wide to clinical staff on development days.

## Author contributions

CT drafted the initial manuscript, PA conceived the project and provided feedback and editing and PC edited the manuscript and provided supervision. PC acts as the guarantor for this article.

## Conflict of interest

None declared.

## Funding

None.
